# Formation and Surface Structures of Long-Range Ordered Self-Assembled Monolayers of 2-Mercaptopyrazine on Au(111)

**DOI:** 10.3390/ijms26010160

**Published:** 2024-12-27

**Authors:** Dongjin Seo, Jin Wook Han, Hongki Kim, Yeon O Kim, Hyun Sun Sung, Riko Kaizu, Glenn Villena Latag, Tomohiro Hayashi, Nam-Suk Lee, Jaegeun Noh

**Affiliations:** 1 Department of Chemistry, Hanyang University, 222 Wangsimni-ro, Seongdong-gu, Seoul 04763, Republic of Korea; nammi72795@hanyang.ac.kr (D.S.); jwhan@hanyang.ac.kr (J.W.H.); audwls8925@naver.com (Y.O.K.); gustjsdl1525@naver.com (H.S.S.); 2 Department of Materials Science and Engineering, School of Materials and Chemical Technology, Institute of Science Tokyo, Yokohama 226-8502, Kanagawa, Japan; kaizu.r.aa@m.titech.ac.jp (R.K.); latag.g.aa@m.titech.ac.jp (G.V.L.); hayashi.t.al@m.titech.ac.jp (T.H.); 3 National Institute for Nanomaterials Technology, Pohang University of Science and Technology, 77 Cheongam-ro, Nam-gu, Pohang 37673, Republic of Korea; 4 Research Institute for Convergence of Basic Science, Hanyang University, 222 Wangsimni-ro, Seongdong-gu, Seoul 04763, Republic of Korea

**Keywords:** 2-mercaptopyrazine, self-assembled monolayers, surface, interface, adsorption structure, scanning tunneling microscopy, X-ray photoelectron microscopy

## Abstract

The effect of solution pH on the formation and surface structure of 2-pyrazinethiolate (2-PyzS) self-assembled monolayers (SAMs) formed by the adsorption of 2-mercaptopyrazine (2-PyzSH) on Au(111) was investigated using scanning tunneling microscopy (STM) and X-ray photoelectron microscopy (XPS). Molecular-scale STM observations clearly revealed that 2-PyzS SAMs at pH 2 had a short-range ordered phase of (2√3 × √21)R30° structure with a standing-up adsorption structure. However, 2-PyzS SAMs at pH 8 had a very unique long-range ordered phase, showing a “ladder-like molecular arrangement” with bright repeating rows. This ordered phase was assigned to the (3 × √37)R43° structure, consisting of two different adsorption structures: standing-up and tilted adsorption structures. The average arial density of 2-PyzS SAMs on Au(111) at pH 8 was calculated to be 49.47 Å^2^/molecule, which is 1.52 times more loosely packed compared to the SAMs at pH 2 with 32.55 Å^2^/molecule. XPS measurements showed that 2-PyzS SAMs at pH 2 and pH 8 were mainly formed through chemical interactions between the sulfur anchoring group and the Au(111) substrates. The proposed structural models of packing structures for 2-PyzS SAMs on Au(111) at different pHs are well supported by the XPS results. The results of this study will provide new insights into the formation, surface structure, and molecular orientation of SAMs by N-heteroaromatic thiols with pyrazine molecular backbone on Au(111) at the molecular level.

## 1. Introduction

Self-assembled monolayers (SAMs) are considered to be effective tools to control the surface and interface properties of various metal and semiconductor substrates, depending on the chemical structure of adsorbates, such as terminal functional groups, molecular backbones, and anchoring groups [1,2,3,4,5,6,7,8,9,10,11,12,13]. These outstanding advantages make SAMs suitable for many practical applications, such as biosensors [14,15], solar cells [16,17], batteries [8], nanopatterning [1], bioelectronics [18], and electronic devices [19,20,21]. In particular, SAMs derived from aromatic and heteroaromatic thiols with π-conjugated systems have attracted much attention due to their interesting electrical properties and controllable charge transfer behaviors in SAM-modified electronic devices [8,22,23]. It has been shown that the physical properties of SAM-modified devices are significantly affected by the structural defects and order of SAMs [24,25] and the orientation of adsorbed molecules [26]. Therefore, it is very important to understand the surface structures of aromatic and heteroaromatic thiolate SAMs from a molecular-scale perspective.

The SAMs on gold surfaces formed by heteroaromatic thiols containing nitrogen atom(s) in the aromatic ring have been studied for understanding the charge transfer behaviors of metalloproteins [27,28,29,30,31], the control of the work function of metal substrates [7,32,33,34,35,36,37], and the electrocatalytic behaviors of metal surfaces [38,39,40,41,42]. Interestingly, heteroaromatic thiol SAMs are often used as primal layers to understand the charge transfer behavior of metalloproteins, such as myoglobin, cytochrome *c*, and ferredoxin [27,28,29,30,31]. It was found that the nitrogen atoms of heteroaromatic thiolate SAMs in contact with the solution interact electrochemically with the positively charged residues of redox proteins. As a result, a fast electron transfer reaction occurs between the redox protein and the metal electrode [29,31]. SAMs derived from 4-mercaptopyridine (4-PySH) and 2-mercaptopyrimidine (2-PymSH) were found to exhibit excellent electron transfer reactions between cytochrome *c* and Au electrodes [29]. However, it is known that the SAMs of 2-mercaptopyridine (2-PySH) do not significantly affect electron transfer reaction [29]. This may be due to the difference in the molecular orientation of heteroaromatic SAMs and the position of nitrogen atoms in the heteroaromatic ring. Scanning tunneling microscopy (STM) measurements were performed to elucidate the formation and molecular-scale surface structures of 2-PyS, 4-PyS, and 2-PymS SAMs on gold surfaces [29,43,44,45]. The 2-PyS SAMs on Au(111) formed in 0.05 M HClO_4_ solution had a *p*(4 × √7)R40.9° packing structure showing a tilted adsorption geometry of the aromatic backbone from the surface normal due to the interaction between the nitrogen atom at position 2 of the heteroaromatic ring and the gold surface; whereas, the 4-PyS SAMs had a *p*(5 × √3)R30° packing structure showing that the molecular backbone is oriented perpendicular to the surface [43]. These results strongly suggest that the molecular self-assembly behavior of heteroaromatic thiols on gold surfaces is significantly affected by the position and number of nitrogen atoms within the heteroaromatic ring, resulting in the formation of different molecular arrangements [43,44,45].

The adsorption structure of SAMs on metal surfaces formed from heteroaromatic thiols containing nitrogen atom(s), such as mercaptopyridine or mercaptopyrimidine derivatives, can be changed by solution pH [44,46,47,48,49]. This is closely related to the protonation and deprotonation processes of nitrogen atom(s) in the heteroaromatic ring and the thiol-thione tautomerization in solution. STM observations revealed that 4-pyridineethanethiolate SAMs on Au(111) in 0.05 M HClO_4_ solution have a *p*(10 × √3)R30° structure; whereas, these SAMs in 0.1 M NaClO_4_ solution have a *p*(5 × √3)R30° structure. This suggests that the structural changes in these SAMs are mainly driven by protonation and deprotonation processes of the molecules [46]. Surface enhanced Raman scatting (SERS) measurements showed that the 2-PyS SAMs on silver surface formed under acidic conditions have a standing-up adsorption orientation; whereas, the 2-PyS SAMs formed under basic conditions have a flat-lying adsorption orientation [47]. High-resolution STM studies revealed that the molecular packing structures of 4-PyS and 2-PymS SAMs on Au(111) formed at high pH, above pH 5, when these molecules exist predominantly in the deprotonated form of nitrogen atom(s) of the heteroaromatic ring, are very similar to each other [44,50]. Meanwhile, N-heteroaromatic thiols exist mainly in the N-protonated form at a low pH below pH 2, as demonstrated in many previous studies [46,47,48,49,50]. For example, 4-PyS SAMs at pH 1 had a (5 × √3)R30° structure; whereas, 4-PyS SAMs at pH 5 and 12 had the same (12 × √3)R30° structure [50]. Additionally, 2-PymS SAMs at pH 2 were mainly composed of a disordered phase; whereas, the formation and structures of 2-PymS SAMs at pH 7 and 12 had the same (4√2 × 3)R51° packing structure [44].

It was reported that the adsorption of 2-mercaptopyrazine (2-PyzSH) on Au(111) resulted in the formation of 2-pyrazinethiolate (2-PyzS) SAMs with very small, ladder-shaped ordered domains containing many structural dislocations [51]. Additionally, 2-PyzS SAMs were found to promote the electron transfer reaction of cytochrome *c* at gold electrode more effectively than 2-PyS SAMs [51]. On the other hand, structural analysis for 2-PyzS SAMs was very difficult due to low STM resolution. To date, there are no molecular-scale STM reports describing the formation and molecular arrangement of 2-PyzS SAMs on Au(111) depending on pH. From a fundamental and practical point of view, it is very important to understand the formation and surface structure of 2-PyzS SAMs by the adsorption of 2-PyzSH molecules on Au(111) at the molecular level.

**Figure 1:**
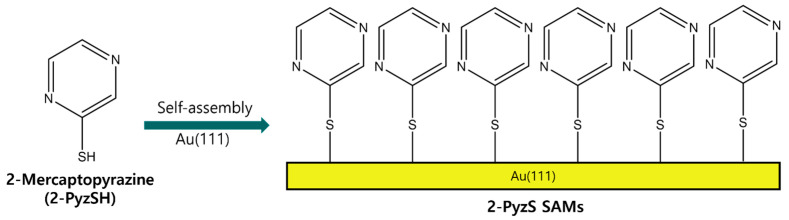
A chemical structure of 2-PyzSH and the formation of 2-PyzS SAMs on Au(111) by the spontaneous adsorption of 2-PyzSH molecules.

Therefore, we examine the formation, surface structure, and adsorption conditions of 2-PyzS SAMs on Au(111), depending on the solution pH using STM and X-ray photoelectron spectroscopy (XPS). Based on the previous STM results showing that N-heteroromatic thiolate SAMs have similar structures at neutral and basic conditions [44,50], we prepared 2-Pyz SAMs on Au(111) at pH 2 and 8 and compared the surface structure and binding conditions of these SAMs at two different pHs to understand how solution pH affects the formation of 2-PyzS SAMs on Au(111). Figure 1 shows the molecular structure of 2-PyzSH and the formation of 2-PyzS SAMs on Au(111) by spontaneous adsorption of 2-PyzSH molecules on a Au(111) surface. In this study, we report the first high-resolution STM results showing the formation and molecular arrangement of 2-PyzS SAMs on Au(111), as well as its pH-dependent phase transition from the (2√3 × √21)R30° structure at pH 2 to the (3 × √37)R43° structure at pH 8.

## 2. Results and Discussion

### 2.1. The Formation and Surface Structures of 2-PyzS SAMs on Au(111) at pH 2

**Figure 2:**
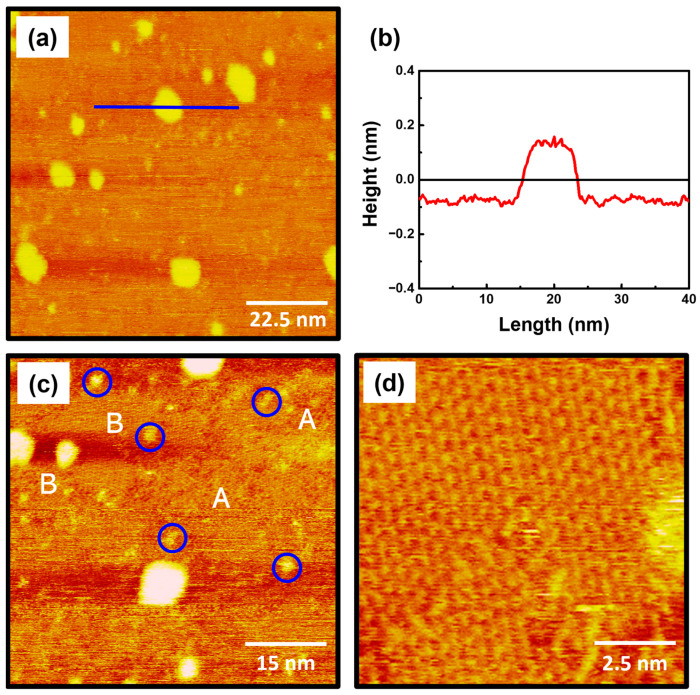
(**a**,**c**,**d**) STM images of 2-PyzS SAMs on Au(111) in 0.01 mM ethanol solution of 2-PyzSH at pH 2 for 1 h at RT. (**b**) Height profile of a blue line drawn along on the bright island protruding from the surface. The scan sizes of the STM images are (**a**) 90 nm × 90 nm, (**b**) 60 nm × 60 nm, and (**d**) 10 nm × 10 nm. The disordered phase, ordered phase, and small bright islands in (**c**) are marked with A, B, and blue circle, respectively.

The STM images in Figure 2 show the surface features of 2-PyzS SAMs on Au(111) formed in 0.01 mM ethanol solution at pH 2 for 2 h at RT. The STM image (90 nm × 90 nm) in Figure 2a shows that the SAMs contain large, bright islands, ranging from a few nm to about 10 nm. Such bright islands have been frequently observed for various SAMs formed from aromatic and heteroaromatic thiols without a methylene spacer between the thiol group and the aromatic (or heteroaromatic) ring [44,52,53,54]. Figure 2b shows the height profile of the blue line drawn along on the bright island in Figure 2a, showing that the bright island, which is 8.5 nm in size, protrudes about 2.4 Å from the SAM surface, which is slightly smaller than the step height of a gold atom with a 2.89 Å. The height of gold adatom islands is typically measured to be around 2.4 Å by STM measurements, so these islands can be assigned to the SAM-covered adatom islands [2,6,52,53,54]. The formation and growth of these islands can be attributed to the slow diffusion rate of SAM-modified Au adatom islands generated during the chemical adsorption of the corresponding thiols, as described in the literature [52]. The STM image (60 nm × 60 nm) in Figure 2c shows that 2-PyzS SAMs consist of three structural features: (a) small bright islands, (b) a disordered phase, and (c) an ordered phase. Many small bright islands of less than 3 nm, indicated by the blue circles, protrude about 1 Å from the surface, which is much smaller than the large bright islands that protrude around 2.4 Å. Therefore, the small bright islands are not SAM-covered Au adatom islands but the molecular aggregates with different adsorption geometry. These surface features were also observed for 2-PymS SAMs on Au(111) formed under various pH conditions [44]. On the other hand, 2-PyzS SAMs were found to be composed of a mixed phase of a predominantly disordered phase (dominant phase, region A) and an ordered phase (region B). Using the same SAM preparation conditions, the adsorption of 2-PymSH molecules on Au(111) was found to produce only a disordered phases [44]. These results demonstrated that the formation and structural arrangements of SAMs of N-heteroaromatic thiols with two nitrogen atoms (2-PymSH and 2-PyzSH) are significantly affected by the position of nitrogen atoms in the heteroaromatic ring. Figure 2d shows an enlarged STM image (10 nm × 10 nm) of the ordered phases (region B, Figure 2c), revealing that these molecules have a well-ordered structure.

**Figure 3:**
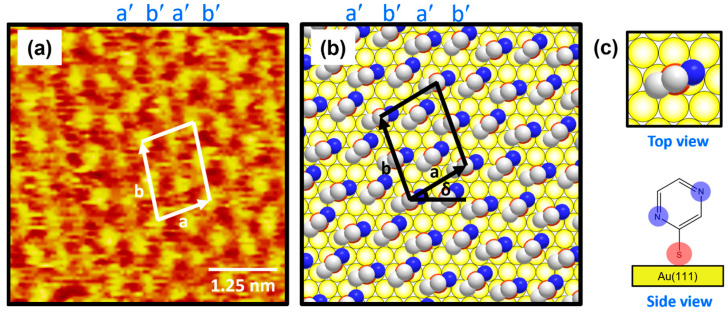
(**a**) High-resolution STM image (5 nm × 5 nm) of 2-PyzS SAMs on Au(111) in 0.01 mM ethanol solution of 2-PyzSH at pH 2 for 1 h at RT. (**b**) Proposed structural model of the SAMs on Au(111). (**c**) Top and side views of 2-PyzS molecules adsorbed on Au(111). In the structural model, white, blue, and red spheres represent the carbon, nitrogen, and sulfur atoms. Note that a and b in (**b**) correspond to the unit cell vectors of lattice, and δ corresponds to rotation angle of the unit cell.

Figure 3a shows the molecularly resolved STM image (6 nm × 6 nm) of the ordered phase for 2-PyzS SAMs on Au(111) at pH 2. The ordered phase has an oblique unit cell and contains alternating molecular rows, appearing as large, bright molecular spots (denoted as a′) and small, bright molecular spots (denoted as b′) in the STM imaging contrast. Based on the high-resolution STM image in Figure 3a, the lattice parameters of the oblique unit cell containing four 2-PyzS molecules adsorbed on Au(111) are extracted: a = 10.0 ± 0.1 Å = 2√3a_h,_ b = 13.2 ± 0.1 Å = √21a_h,_ and δ = 30°, where a_h_ is the interatomic distance between the Au atoms (2.89 Å), as shown in Figure 3b. This ordered phase is assigned to the (2√3 × √21)R30° packing structure. The structural model for this ordered phase is proposed in Figure 3b, and the top and side views of the 2-PyzS molecule adsorbed on the Au(111) surfaces are shown in Figure 3c. Considering the XPS results showing a weak intensity of N 1s peak arising from the interactions between the nitrogen atoms of the pyrazine ring and the Au(111) surface (discussed in detail later in the XPS section), all 2-PyzS molecules in the SAMs are predicted to have a standing-up molecular orientation, as shown in the structural model in Figure 3b. It is suggested that the two different molecular spots within the unit cell are due to differences in the adsorption geometry due to changes in the rotation angle of the adsorbed molecules (molecular rows labeled b′) to minimize the repulsion between the pyrazine molecules in the SAMs. The average arial density of 2-PyzS SAMs on Au(111) was calculated to be 32.55 Å^2^/molecule, which is almost the same as that of benzenethiolate SAMs with 34.81 Å^2^/molecule [53]. This suggests that, when 2-PyzSH molecules are adsorbed on Au(111) at pH 2, a relatively dense monolayer similar to benzenethiol without nitrogen atoms in the aromatic ring is formed.

### 2.2. The Formation and Surface Structures of 2-PyzS SAMs on Au(111) at pH 8

**Figure 4:**
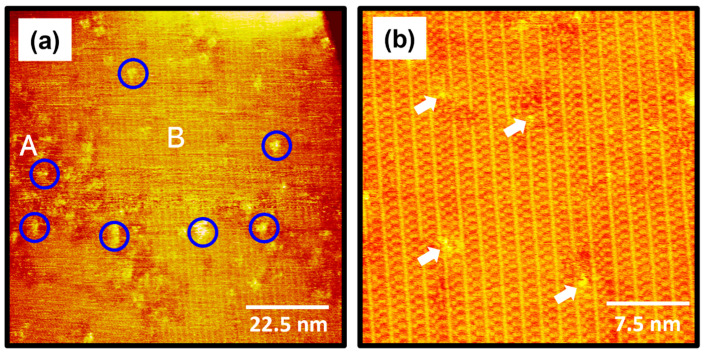
(**a**,**b**) STM images of 2-PyzS SAMs on Au(111) in 0.01 mM ethanol solution of 2-PyzSH at pH 8 for 1 h at RT. The scan sizes of the STM images are (**a**) 90 nm × 90 nm and (**b**) 30 nm × 30 nm. The disordered phase, ordered phase, and small bright islands in (**a**) are marked with A, B, and blue circle, respectively. Structural defects in (**b**) are indicated by the white arrows.

The STM images in Figure 4 show the surface structures of 2-PyzS SAMs on Au(111) formed in a 0.01 mM ethanol solution at pH 8 for 1 h at RT. STM observations clearly reveal that the formation and molecular arrangements of SAMs formed at pH 8 are completely different from those of SAMs formed at pH 2 (Figure 2). The STM image (90 nm × 90 nm) in Figure 4a shows that, although numerous Au adatom islands (large bright islands shown in Figure 2) appeared at pH 2 during 2-PyzS SAM formation, no such islands were observed when SAMs were formed at pH 8. These results suggest that the diffusion rate of SAM-covered Au adatoms generated by the adsorption of 2-PyzSH molecules at pH 8 is much faster than that of the SAM-covered Au adatoms generated by the adsorption of 2-PyzSH molecules at pH 2. The 2-PyzS SAMs at pH 8 have three structural characteristics: (a) small bright islands (indicated by blue circles), (b) a disordered phase (region A), and (c) an ordered phase (region B), as in the case of SAMs at pH 2. However, unlike the SAMs at pH 2, the ordered phase is predominantly formed on the entire Au surface; whereas, the disordered phase is partially present. Interestingly, the adsorption of 2-PyzSH molecules on Au(111) at pH 8 results in the formation of long-range ordered SAMs with unidirectional ordered domains typically larger than about 90 nm, as shown in Figure 4a. These results can be attributed to the faster diffusion rate of deprotonated 2-PyzSH molecules on Au(111) at pH 8 during SAM formation compared to that of protonated 2-PyzSH molecules at pH 2, resulting in the formation of large, ordered domains. These results can be supported by numerous previous studies showing that the structural order of organic thiolate SAMs with large domains is significantly enhanced when the diffusion rate of thiol molecules on Au(111) increases with increasing deposition temperature [1,2,4,7,52,53]. The high-resolution STM image (30 nm × 30 nm) in Figure 4b shows well-ordered 2-PyzS SAMs on Au(111) with a large, ordered domain. Structural defects are present in the SAMs with bright contrast in the ordered domains indicated by white arrows, which are probably due to a metastable orientation of the heteroaromatic ring and/or the metastable adsorption condition of the sulfur anchoring group on Au(111) in the SAMs. These molecular defects were also observed in the SAMs formed by the adsorption of dodecyl thiocyanates at 50 °C [55].

**Figure 5:**
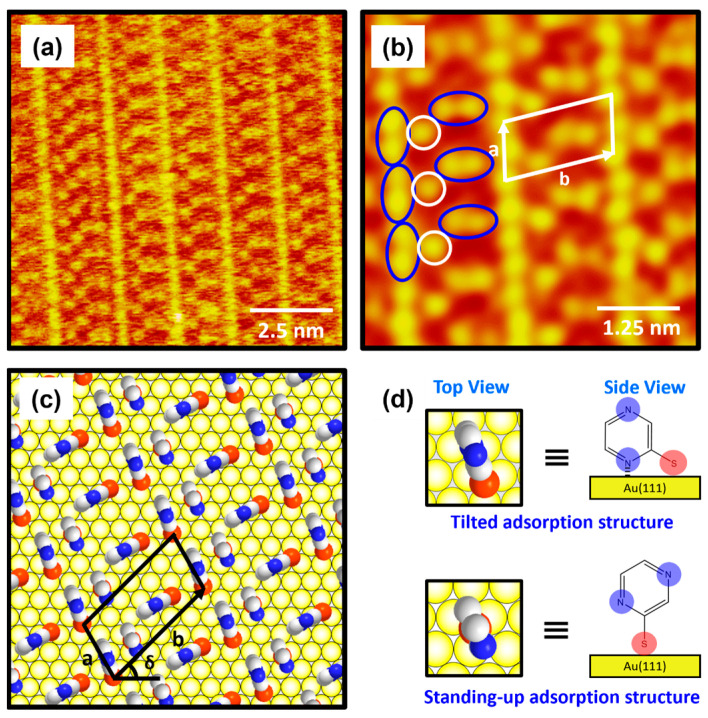
(**a**) High-resolution STM image and (**b**) low-pass-filtered STM image of 2-PyzS SAMs on Au(111) in 0.01 mM ethanol solution of 2-PyzSH at pH 8 for 1 h at RT. (**c**) Proposed structural model of the SAMs on Au(111). (**d**) Top and side views of 2-PyzS molecules adsorbed on Au(111). In the structural model, white, blue, and red spheres represent the carbon, nitrogen, and sulfur atoms. The scan sizes of the STM images are (**a**) 10 nm × 10 nm and (**b**) 5 nm × 5 nm. The one and paired molecular spots in (**b**) are marked with open white and oval blue circles, respectively. Note that a and b in (**b**,**c**) correspond to the unit cell vectors of lattice, and δ in (**c**) corresponds to rotation angle of the unit cell.

The molecularly resolved STM image (10 nm × 10 nm) in Figure 5a clearly shows that 2-PyzS SAMs at pH 8 contained very unique, well-ordered domains that have never been observed in SAMs formed from other aromatic and N-heteroaromatic thiols on Au(111). The low-pass-filtered, enlarged STM image (6 nm × 6 nm) in Figure 5b shows that the ordered phase has a very unique long-range ordered phase showing a “ladder-like molecular arrangement” with bright repeating rows and has an oblique unit cell containing two different molecular features: one molecular spot (open circle in white) and a paired molecular spot (oval circle in blue). Based on the high-resolution STM images in Figure 5a,b, the lattice parameters of the oblique unit cell containing three 2-PyzS molecules adsorbed on Au(111) are deduced: a = 9.0 ± 0.2 Å = 3a_h_, b = 17.4 ± 0.2 Å = √37a_h_, and δ = 43°, where a_h_ is the interatomic distance between the Au atoms. This ordered phase can described as the (3 × √37)R43° packing structure, which is completely different from the packing structures of SAMs formed by other N-heteroaromatic thiols, such as 2-PySH, 4-PySH, and 2-PymSH [29,43,44]. Figure 5c shows the proposed structural model of 2-PyzS SAMs on Au(111) containing two different adsorption geometries in the unit cell, and the top and side views of 2-PyzS molecules corresponding to two adsorption geometries are shown in Figure 5d. The 2-PyzS molecule, showing one molecular spot in STM imaging (open circle in white), is predicted to have a standing-up molecular orientation; whereas, the 2-PyzS molecule, showing a paired molecular spot (oval circle in blue), is predicted to have a tilted molecular orientation as a result of interactions between the nitrogen atom nearest to the S anchoring group in the pyrazine ring and the Au(111) surface (this is supported by the presence of N 1s XPS peak with a strong intensity due to these nitrogen atoms and is discussed in detail in the XPS section). Interestingly, although the packing structures of 2-PyS and 2-PymS SAMs on Au(111) are completely different, “ladder-shaped molecular arrangement” is commonly observed [29,43,44,45], as can also be seen in the current study on 2-PyzS SAMs. Such a ladder-shaped molecular arrangement can only be found in the SAMs of N-heteroaromatic thiols, where the nitrogen atom is attached at the position 2 with respect to the SH group. Therefore, it is reasonable to consider that this unique structure for 2-PyzS SAMs is mainly due to the interactions between the 2-position nitrogen atom in the pyrazine ring and the Au(111) surface, resulting in the formation of SAMs with a tilted adsorption structure, as shown in Figure 5d. The average arial density of 2-PyzS SAMs on Au(111) at pH 8 was calculated to be 49.47 Å^2^/molecule, which is 1.52 times more loosely packed compared to the SAMs at pH 2 with 32.55 Å^2^/molecule. The packing structure and average arial molecular density of 2-PyzS SAMs on Au(111) at pH 2 and 8 are summarized in Table 1. The formation of this loosely packed monolayer at pH 8 is due to the fact that most pyrazinethiolate molecules in the SAMs prefer to have a tilted adsorption structure. This may be possible, because most of the nitrogen atoms in the 2-PyzS molecules in solution at pH 8 exist in the deprotonated form. In this study, we report the first high-resolution STM results showing the formation and molecular arrangement of 2-PyzS SAMs on Au(111) and the pH-dependent structural change from the (2√3 × √21)R30° structure at pH 2 to the (3 × √37)R43° structure at pH 8.

### 2.3. S 2p XPS Peaks for 2-PyZS SAMs on Au(111) at Different Solution pH

**Figure 6:**
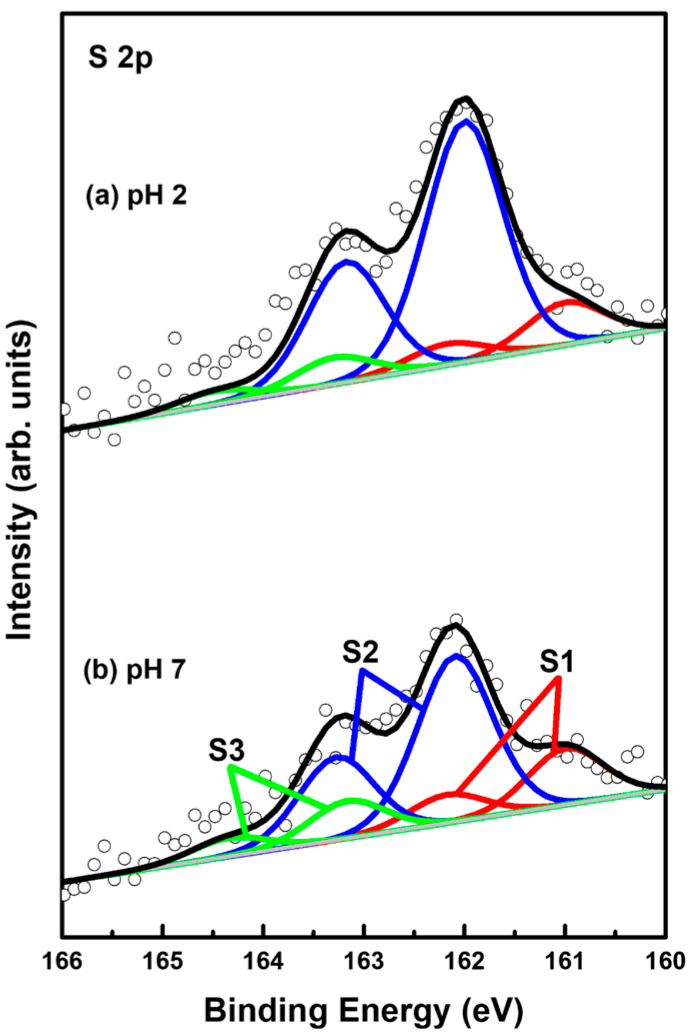
S 2p XPS spectra of 2-PyzS SAMs on Au(111) in 0.01 mM ethanol solution of 2-PyzSH at (**a**) pH 2 and (**b**) pH 8 for 1 h at RT.

XPS measurements can provide very useful information about the surface and interfacial properties of organic monolayers on metal surfaces [2,4,9,32,55,56,57,58,59,60,61]. Therefore, the adsorption conditions of sulfurs in 2-PyzS SAMs on Au(111) formed at pH 2 and pH 8 conditions were investigated by XPS to understand the formation and adsorption structures of SAMs. Figure 6 shows the S 2p XPS peaks of the 2PyzS SAMs on Au(111) formed at (a) pH 2 and (b) pH 8. In principle, the S 2p peak appears as a doublet consisting of 2p_1/2_ and 2p_3/2_ components in a 1:2 intensity ratio due to spin-orbital splitting [2,4,33,55,56,57,58,59]. The XPS results in Figure 6 show that 2-PyzS SAMs at pH 2 and pH 8 have three S 2p peaks, labeled S1 (red), S2 (blue), and S3 (green), which means the existence of three different sulfur adsorption conditions in the SAMs. The binding energies of S 2p_3/2_ components for the S1, S2, and S3 peaks are summarized in Table 2. It is known that the S1 peak observed around 161 eV and the S2 peaks observed around 162 eV for organic thiolate SAMs are due to bound sulfurs (chemisorbed species) on the gold surface; whereas, the S3 peak observed around 163 eV is due to unbound sulfurs (physisorbed species), as elucidated by many previous studies [2,4,33,55,56,57,58,59]. In particular, the bound S1 peak is generally observed in organic thiolate SAMs with loosely packed and disordered phases; whereas, the bound S 2p peak often appears as the predominant peak, when thiolate SAMs have closely packed and well-ordered domains [55,56,57]. On the other hand, the unbound S3 peak is often observed around 163–164 eV in various SAM samples. The origin of these unbound sulfur species may be due to the presence of physically adsorbed molecules on the surface and/or the presence of unreacted thiols trapped inside the SAMs during SAM formation [58]. In these XPS measurements, the bound S2 peak is observed as the main peak of 2-PyzS SAMs at both pH 2 and pH 8.

The XPS relative intensities of bound sulfurs (S1 + S2) against Au 4f for 2-PyzS SAMs on Au(111) at pH 2 and pH 8 are summarized in Table 2. The relative intensities of sulfurs (S1 + S2)/Au 4f were measured to be 0.00182 (pH 2) and 0.00129 (pH 8), respectively. This means that the surface coverage of 2-PyzS SAMs chemisorbed on Au(111) at pH 2 was significantly increased by 41% compared to that of 2-PyzS SAMs at pH 8. These results are in good agreement with the STM results. In addition, the relative intensities of physisorbed sulfur (S3) to the chemisorbed sulfur (S1 + S2) were measured to be 0.093 (9.3%, pH 2) and 0.163 (16.3%, pH 8), implying that 2-PyzS SAMs are mainly formed via the chemical reactions between the SH anchoring group of pyrazine molecules and the Au(111) surface.

### 2.4. N 1s XPS Peaks for 2-PyZS SAMs on Au(111) at Different Solution pH

**Figure 7:**
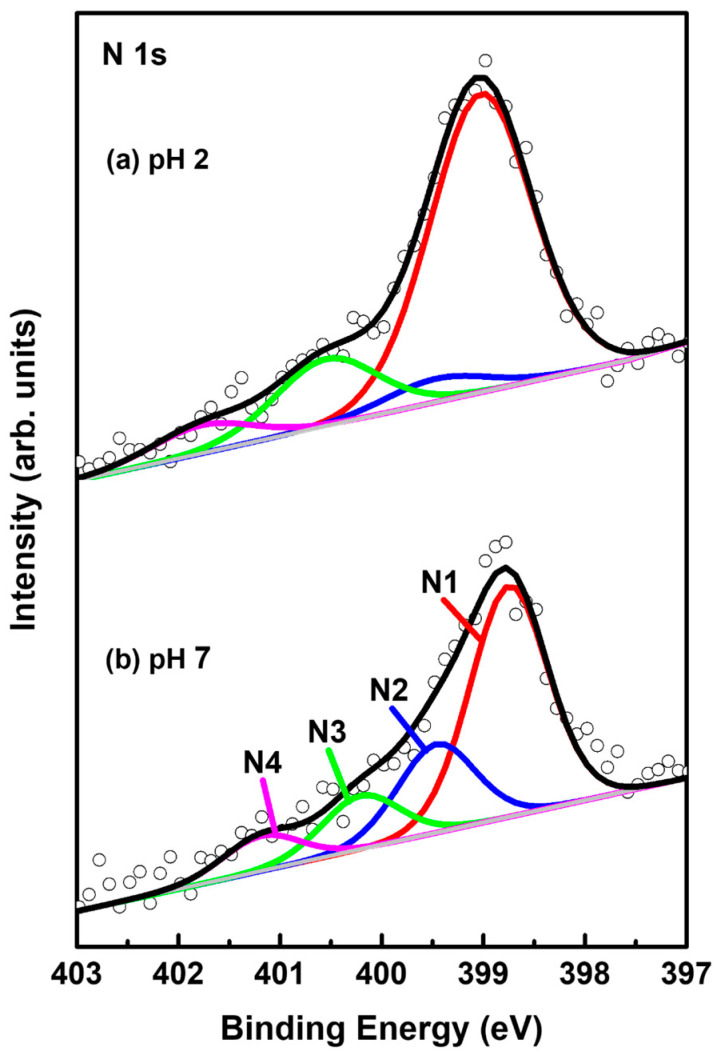
N 1s XPS spectra of 2-PyzS SAMs on Au(111) in 0.01 mM ethanol solution of 2-PyzSH at (**a**) pH 2 and (**b**) pH 8 for 1 h at RT.

Figure 7 shows N 1s XPS peaks of 2-PyzS SAMs on Au(111) at (a) pH 2 and (b) pH 8. The N 1s spectrum was separated into four peaks, labeled as N1 (red), N2 (blue), N3 green), and N4 (pink), implying the existence of four different nitrogen species in the SAMs. These four N1 XPS peaks were also observed for 2-PymS monolayers adsorbed on gold nanoparticles [59] and on Au(111) surfaces [44]. These results imply that the adsorption structures of SAMs formed by 2-PymSH and 2-PyzSH molecules containing two nitrogen atoms in the pyrimidine and pyrazine rings are very complicated. STM results also showed that the surfaces of these SAMs were inhomogeneous, consisting of several structural features (adatom islands, bright molecular aggregates, disordered phase, and ordered phase), as discussed in the STM section (Figure 2 and Figure 4). The binding energies of N1, N2, N3, and N4 peaks and the XPS relative intensities of (N1 + N2)/Au 4f for 2-PyzS SAMs at pH 2 and pH 8 are summarized in Table 3. The intensity of the N1 peak appearing around 398.9 eV was found to be very strong compared to other N peaks. This peak is attributed to the deprotonated N atoms of the pyrazine ring, while the N2 peak, appearing at approximately 399.6 eV, is attributed to the N atoms of the pyrazine ring interacting with the Au(111) surface, as suggested in previous studies [60,61]. The structural model regarding these adsorption structures can be clearly seen in Figure 5d. On the other hand, the N3 and N4 peaks appearing around 400.6 eV and 401.5 eV are attributed to the N atoms in different degrees of H acceptance and the N-protonation of the pyrazine ring in the SAMs [60,61]. The most notable difference between N 1s XPS peaks for the two SAM samples is that the relative intensity of N1/N2 peak is significantly different from each other: that of N1/N2 peak for 2-PyzS SAMs at pH 2 was found to be 12.96; whereas, that of N1/N2 peak for 2-PyzS SAMs at pH 8 was found to be 2.61. These results strongly suggest that most nitrogen atoms of 2-PyzS molecules in the SAMs on Au(111) at pH 2 dominantly existed in the deprotonated form, resulting in the formation of SAMs with a standing-up adsorption structure, as revealed by this STM study (Figure 3). On the other hand, the nitrogen atoms of 2-PyzS molecules in the SAMs on Au(111) at pH 8 existed in two types: one is a nitrogen atom in the deprotonated form, and the other is a nitrogen atom interacting with Au(111) surface, resulting in the formation of SAMs with a tilted adsorption structure, as suggested by this STM study (Figure 5). Interestingly, the relative intensity of nitrogen atoms involved in H acceptance and protonation (N3 + N4) to the total nitrogen atoms (N1 + N2 + N3 + N4) in the SAMs on Au(111) is calculated to be approximately 0.22 (22%) regardless of the solution pH. However, the relative intensity of the N3 + N4 peaks to the N2 peak is completely dependent on the solution pH. That is, this relative intensity of SAMs at pH 2 was found to be 4.12, while that of SAMs at pH 8 was found to be 1.06. This means that the relative intensity of the N3 + N4 peaks to the N2 peak at pH 2 is 3.9 times stronger than at pH 8. Therefore, it is considered that 2-PyzS molecules in the SAMs at pH 2 prefer nitrogen atoms to exist in the N-protonated form (intermolecular hydrogen bonding) and also to exist partially interacting with H^+^ atoms rather than interacting with the Au(111) surface. In contrast, the nitrogen atoms of 2-PyzS molecules at pH 8 prefer to interact with the Au(111) surface. Based on high-resolution STM images, the proposed structural models of the packing structures for 2-PyzS SAMs on Au(111) at different pHs are well supported by the XPS results.

## 3. Materials and Methods

### 3.1. Chemicals and Fabrication of Au(111) Substrates

The 2-PyzSH (purity ≥ 98%) was purchased from Tokyo Chemical Industry Co., LTD. (Tokyo, Japan) Single-crystal Au(111) substrates with large atomically flat large terraces in the range of 100–400 nm were fabricated by thermal evaporation of gold onto a freshly cleaved mica surface preheated to 570 K under vacuum conditions of approximately 10^−5^ Pa [4].

### 3.2. Preparation of 2-PyzS SAMs

The 2-PyzS SAMs were prepared by soaking clean Au(111)/mica substrates in a 0.01 mM ethanol–water (95:5 wt%) solution of 2-PyzSH at pH 2 and pH 8 for 1 h at RT. Note that when a pure water solvent was used to prepare 2-PyzS SAMs on Au(111), the Au(111) film peeled off from mica sheet during SAM formation. Therefore, to avoid this problem, we prepared a pH 2 solution using a cosolvent system of ethanol–water (95:5 wt%). A pH 2 solution was prepared by dropping 1 M HCl or 1 M NaOH solutions using a micro syringe into the ethanol–water (95:5 wt%) solution of 2-PyzSH using a pH meter. The pH of 0.01 mM ethanol–water solution containing 2-PyzSH was measured to be almost pH 8. The prepared SAM samples were thoroughly rinsed with ethanol to remove 2-PyzSH molecules physisorbed on the surface and dried under a high-purity N_2_ gas flow.

### 3.3. STM and XPS Measurements

STM measurements were performed with a NanoScope E (Veeco, Santa Barbara, CA, USA) with a commercially available Pt/Ir (80:20) tip. All STM images were acquired in constant current mode using a bias voltage ranging from 300 to 500 mV and a tunneling current ranging from 300 to 600 pA between the tip and SAM samples. XPS measurements were carried out using a K-alpha plus system (Thermo Scientific, Waltham, MA, USA) with a monochromatic Al K_α_ radiation source (1486.6 eV). The resulting XPS spectra were calibrated based on the binding energy of 84.0 eV for Au 4f_7/2_ peak (84.0 eV). A curve fitting analysis using Voigt functions was conducted to determine the positions of doublet 2p_1/2_ and 2p_3/2_ sulfur 2p peaks.

## 4. Conclusions

The surface structures and adsorption conditions of 2-PyzS SAMs on Au(111) as a function of the solution pH were investigated using STM and XPS. The STM observations revealed that the formation and molecular packing structure of the SAMs at pH 2 were completely different from those formed at pH 8. The 2-PyzS SAMs at pH 2 were composed of several structural features: (a) large Au adatom islands, (b) small bright molecular aggregates, (c) a disordered phase as a dominant phase, and (d) a short-range ordered phase; whereas, 2-PyzS SAMs at pH 8 consisted of different structural features: (a) small bright molecular aggregates, (b) a disordered phase, and (c) a long-range ordered phase as a dominant phase. High-resolution STM imaging showed that the ordered phase of 2-PyzS SAMs at pH 2 was assigned to the (2√3 × √21)R30° structure with a standing-up adsorption structure; whereas, 2-PyzS SAMs at pH 8 had an unidirectional, long-range ordered phase, with the (3 × √37)R43° packing structure consisting of two different adsorption structures: standing-up and tilted adsorption structures. XPS measurements showed that 2-PyzS SAMs at pH 2 and pH 8 were mainly formed through chemical interactions between the sulfur anchoring group and the Au(111) substrates. The surface coverage of 2-PyzS SAMs on Au(111) at pH 2 was increased by 41% compared to that of 2-PyzS SAMs at pH 8. The proposed structural models of packing structures for 2-PyzS SAMs on Au(111) at different pHs are well supported by the XPS results. The results of this study can provide very meaningful information about the formation, surface structure, and molecular orientation of pyrazinethiol on Au(111) depending on the solution pH.

## Figures and Tables

**Table 1:** Packing structure and average arial molecular density of 2-PyzS SAMs on Au(111) at pH 2 and pH 8.

SAM Sample	pH Condition	Packing Structure	Arial Molecular Density
2-PyzS SAMs	2	(2√3 × √21)R30°	32.55 Å^2^/molecule
2-PyzS SAMs	8	(3 × √37)R43°	49.47 Å^2^/molecule

**Table 2:** XPS peaks in the S 2p region of 2-PyzS SAMs on Au(111) and XPS relative intensities of S 2p/Au 4f and of bound sulfurs (S1 + S2)/Au 4f at different solution pHs.

pH Condition	S 2p Species	Peak (eV) ^a^	S 2p/Au 4f ^b^	S1 + S2/Au 4f ^b^
pH = 2	S1	161.05	0.00027	0.00182
	S2	162.06	0.00152	
	S3	163.30	0.00017	
pH = 7	S1	161.11	0.00041	0.00129
	S2	162.13	0.00088	
	S3	163.17	0.00021	

^a^ S 2p: S 2p_3/2_ only; ^b^ Au 4f: Au 4f_7/2_.

**Table 3:** XPS peaks in the N 1s region of 2-PyzS SAMs on Au(111) and XPS relative intensities of N 1s/Au 4f and deprotonated nitrogen (N1 + N2)/Au 4f at different solution pHs.

pH Condition	N 1s Species	Peak (eV)	N 1s/Au 4f ^a^	N1/N2
pH = 2.0	N1	398.98	0.00324	12.96
	N2	399.52	0.00025	
	N3	400.55	000073	
	N4	401.77	0.00030	
pH = 8	N1	398.95	0.00175	2.61
	N2	399.66	0.00067	
	N3	400.41	0.00041	
	N4	401.38	0.00027	

^a^ Au 4f: Au 4f_7/2_.

## Data Availability

The data presented in this study are available in this article.
